# Supplementary material for: Transfer Learning and Permutation‐Invariance Improving Predicting Genome‐Wide, Cell‐Specific and Directional Interventions Effects of Complex Systems
Source: Adv Sci (Weinh). 2025 Sep 19;12(46):e09456. doi: 10.1002/advs.202509456 (PMC12697789; doi:10.1002/advs.202509456)
Supplement: Supplementary file 1 — Supporting Information [file ADVS-12-e09456-s001.docx]

Supplementary materials for

Transfer Learning and Permutation-Invariance improving Predicting Genome-wide, Cell-Specific and Directional Interventions Effects of Complex Systems

Boyang Wang^a^, Pan Boyu^b^, Tingyu Zhang^a^, Qingyuan Liu^a^, Shao Li^a,^*

^a^ Institute for TCM-X, Department of Automation, Tsinghua University, Beijing 100084, China.

^b^ Department of Molecular Pharmacology, Tianjin Medical University Cancer Institute & Hospital; National Clinical Research Center for Cancer; Key Laboratory of Cancer Prevention and Therapy, Tianjin; Tianjin's Clinical Research Center for Cancer, Tianjin 300060, China.

***Address correspondence to:**

Prof. Shao Li, Institute for TCM-X, Department of Automation, Tsinghua University, Beijing 100084, China. E-mail: [shaoli@mail.tsinghua.edu.cn](mailto:shaoli@mail.tsinghua.edu.cn)

**Methods and materials**

**Construction and training of the SETComp model**

**Training details for pre-training and fine-tuning of the model**

Our goal is to predict the up- or down-regulatory effect of given compounds or NP on every gene of a certain cell line. So, the model is trained based on a common classification loss with L2 regulation. Given the total number of training samples $N$, and the number of classes in the classification task $K=3$, for each sample, $y_{ij}$ is the ground-truth label for class $j$ and $\hat{y}_{ij}$ is the predicted probability for class $j$:

$$L=-\frac{1}{N}\sum_{i=1}^{N} \sum_{j=1}^{K} y_{ij}log\hat{y}_{ij}+\lambda||\theta||_{2}^{2}$$

In the pre-training procedure, 28,627,074 compound-cell line-gene pairs, in which compound served as chemical compositions in at least one natural product, were extracted as the test dataset. To address the potential issue of imbalance among samples, we applied a down-sampling method, reducing the sample sizes of the other two categories to match the size of the smallest category. And the training and validation sets were divided in a 7:3 ratio. Input features of training data, validation data and test data were scaled on training data for compounds and genes, respectively. Grid search was applied to find the best hyper-parameter combination, in which the search ranges were set as follows: batch size: 1024, 512, 256, 128, 64; learning rate: 1e-3, 1e-4, 1e-5, 1e-6; L2 regularization: 1e-5, 1e-4, 1e-3, 1e-2; dropout: 0.1, 0.2, 0.5, 0.8; dimension in MLP layer: 512, 1024; dimension in Set Transformer layer: 512, 1024, 2048. For each combination, the model was trained for 5 epochs. And for the add version, we used the same structure for grid search, while L2 regularization and dropout were set as the varied parameters: L2 regularization: 1e-5, 1e-4, 1e-3, 1e-2; dropout: 0.1, 0.2, 0.5, 0.8.

In the fine-tuning step, the down-sampling method was also performed, while the training, validation and test sets were divided in a 7:2:1 ratio. Input features of training data, validation data and test data were scaled on training data for compounds and genes, respectively. Another grid search was performed, in which the search ranges were set as follows: batch size: 1024, 512, 256, 128, 64; learning rate: 1e-3, 1e-4, 1e-5, 1e-6; L2 regularization: 1e-5, 1e-4, 1e-3, 1e-2; dropout: 0.1, 0.2, 0.5, 0.8. For each combination, the model was trained for 50 epochs with an early stopping strategy, setting patience as 5 epochs.

Each compound in the NP was embedded using our trained compound embedding module (which includes the 300-dimensional vector derived from the trained InfoGraph^1^ model and the 881-dimensional vector based on PubChem's fingerprint representation). Then, the representations of each compound were concatenated along the sequence length to form a set vector of length m and 1081 dimensions (881 dimensions + 300 dimensions), where m is the number of compounds in the NP. All NP set representations were then padded to a length of 512, with the regions not containing compounds being masked using mask.

The model was trained in two 4090 GPUs with DataParallel implemented in PyTorch.

**Main architecture of the model**

The core idea of Deep Sets is to encode a set $X$ by applying a shared transformation $\varphi$ to each element and then aggregating the transformed elements using a permutation-invariant operation $\rho$ such as summation or averaging. Mathematically, this can be expressed as:

$$X=\{x_{1},x_{2},\ldots,x_{n}\}$$

$$f\left( X \right)=\rho(\left\{ \varphi\left( x_{1} \right), \varphi\left( x_{2} \right), \ldots,\varphi(x_{n}) \right\})$$

This formulation ensures that the output representation $f(X)$remains unchanged under any permutation of the input set elements, capturing the essence of the set's properties without imposing any arbitrary ordering.

Building upon the foundation of Deep Sets, Set Transformer introduce a more expressive and scalable approach by incorporating self-attention mechanisms, inspired by the success of transformers in sequence modeling. Set Transformer are designed to handle sets by using attention layers that are permutation-invariant and can model complex interactions between set elements.

The Multihead Attention Block (MAB) is the core component of the Set Transformer. MAB combines queries $Q$, keys $K$, and values $V$, leveraging the multi-head self-attention mechanism to capture relationships between elements. It is defined as:

$$MAB\left( Q,K,V \right)=LN(Q+MultiHead\left( Q,K,V \right))$$

where$LN$denotes Layer Normalization, and$MultiHead$represents the multi-head attention mechanism, computed as:

$$MultiHead\left( Q,K,V \right)=Concat\left( head_{1},\ldots,head_{h} \right)W^{O}$$

Each attention head $head_{i}$ is calculated as:

$$head_{i}=Attention(QW_{i}^{Q},KW_{i}^{K},VW_{i}^{V})$$

The Set Attention Block (SAB) is composed of MABs and is used to perform self-attention operations on the input set. SAB is formulated as:

$$SAB\left( X \right)=MAB(X,X,X)$$

where $X$ represents the input set's representation. Through SAB, the model can capture higher-order interactions among set elements.

The Induced Set Attention Block (ISAB) introduces the concept of inducing points to reduce computational complexity. ISAB uses a fixed number of inducing points to map the input set to a lower-dimensional space, thereby reducing computation while maintaining model performance. It is defined as:

$$H=MAB\left( I,X \right)$$

$$ISAB\left( X \right)=MAB(X,H)$$

where $I$ is the learnable inducing point matrix, and $H$ is the intermediate representation.

Pooling by Multihead Attention (PMA) is used to aggregate variable-length set representations into a fixed-size global representation. PMA employs a set of $k$ learnable seed vectors, interacting with the input set through the multi-head attention mechanism, defined as:

$$PMA_{k}(X)=MAB(S,X)$$

where $S$ is a set of $k$ learnable seed vectors, and $k$ is the desired number of output elements.

By stacking these attention blocks, Set Transformer captured higher-order interactions among elements without assuming any particular sequence, making them highly suitable for tasks involving set inputs. The self-attention mechanism allows the model to dynamically weigh the relevance of each element relative to others, resulting in a richer representation of the set. In general, we leveraged these architectures to process inputs naturally represented as sets, ensuring that our models remained invariant to the permutation of input elements while effectively capturing complex relationships within the data. This approach enhances the model's ability to generalize and accurately reflect the underlying structure of the data without introducing biases associated with input ordering.

**Baseline model comparison and ablation studies**

In comparison to our proposed model in both predicting the targets of single compounds and NP, we applied baseline models for pre-training and fine-tuning, respectively. For pre-training procedure, we trained classic machine learning models including K-Nearest Neighbors (KNN) and Decision Tree (DT), Linear Discriminant Analysis (LDA), as well as deep learning models for ablation studies, including Vanilla Neural Network (MLP), MLP with attention module (named No-set), and model with same structures but trained on smaller training datasets. KNN and DT were constructed based on python package scikit-learn. As in the pre-training procedure, embeddings of compounds were sets containing only one element, so we treated them as 2D vectors which would be directly combined with the embeddings of genes and cell lines as the input features for No-set, MLP, KNN, and DT. Besides, we also trained models on smaller dataset which had 1/100 training samples of the original training dataset. This was done on the Concat version, Add version of our models, as well as No-set, MLP, KNN, and DT. We only test the performance of KNN and DT on the smaller dataset for their low computing efficiency and large demand of memories. In general, in the pre-training step, we directly compared the full version of the Concat version, Add version of our model with No-set on the full training dataset, as well as smaller version of the Concat version, Add version of our model with No-set, MLP, KNN, and DT on the 1/100 dataset. In details, the training parameters for MLP and No-set chose the best grid search parameters of the Concat version in the pre-training grid search.

In fine-tuning procedure, we compared the performance of the Concat version, Add version of our model with No-set, MLP, KNN, DT, and LDA directly trained on the fine-tuning dataset as well as small pre-training dataset with fine-tuning dataset, MLP and No-set trained on pre-training dataset with fine-tuning dataset. Additionally, we also compared our models with the Concat version, Add version of our models only trained on the pre-training dataset, only trained on the fine-tuning dataset, as well as the models trained on the smaller pre-training dataset and full fine-tuning dataset. Specially, as No-set, MLP, KNN, DT, LDA only accepted 2D dimension feature in this circumstance, the set input was averaged into 2D dimension vector within their training or testing. Further we estimated the performance of our models which were trained on the full pre-training dataset and different percentages of the fine-tuning dataset to find the relationship between the performance and the size of the fine-tuning dataset. In details, the training parameters for MLP and No-set chose the best grid search parameters of the Concat version in the fine-tuning grid search. Finally, we also tried another fine-tuning task, in which we divided the whole fine-tuning datasets according to the intervention of different NP. Among the 143 kinds of NP, transcriptomics intervened by 10% of them (14) were chosen as the test data, and the remaining part was split into the training set and the validation set with the ratio of 7:3. The grid search was also performed, in which the search ranges were set as follows: batch size: 1024, 512, 256, 128, 64; learning rate: 1e-3, 1e-4, 1e-5, 1e-6; L2 regularization: 1e-5, 1e-4, 1e-3, 1e-2; dropout: 0.1, 0.2, 0.5, 0.8.

In the comparison to baseline models and the ablation studies, Area under ROC curve (AUC), Area under Precision-Recall curve (AUPR), Accuracy (Acc) and F1 score were chosen as the metrics for comparison. Acc was calculated according to:

$$Acc=\frac{1}{N}\sum_{i=1}^{N} 1(\hat{y}_{i}=y_{i})$$

F1 score was weighted-average, for every class $i$:

$$F1_{i}=\frac{2\cdot Precision_{i}\cdot Recall_{i}}{Precision_{i}+Recall_{i}}$$

$$F1_{weighted}=\frac{\sum_{i=1}^{K} N_{i}\cdot F1_{i}}{N}$$

In which:

$$Precision_{i}=\frac{TP_{i}}{TP_{i}+FP_{i}}$$

$$Recall_{i}=\frac{TP_{i}}{TP_{i}+FN_{i}}$$

And $TP_{i}$ is the number of true-positive for class $i$, $FP_{i}$ is the number of false-positive for class $i$ and $FN_{i}$ is the number of false-negative for class $i$.

AUC was also weighted-average on every class:

$$AUC_{i}=\int_{0}^{1} TPR_{i}\left( FPR \right)dFPR$$

$$AUC_{macro}=\frac{1}{K}\sum_{i=1}^{K} AUC_{i}$$

So as AUPR:

$$AU{PR}_{i}=\int_{0}^{1} Precision_{i}\left( Recall \right)dRecall$$

$$AU{PR}_{macro}=\frac{1}{K}\sum_{i=1}^{K} AU{PR}_{i}$$

**In vitro cell lines intervention by multiple NP for validation**

**Quality control and alignment**

We collected 13 NPs (with each NP containing ≥ 50 compounds), for which we had available samples, and randomly selected three of these NPs. The raw data in FASTQ format were first processed using a Perl script to remove adapter sequences and filter out low-quality reads (where the number of bases with quality scores ≤ 25 exceeds 60% of the total reads) and reads with more than 5% N (N indicates undetermined base information), resulting in clean reads for subsequent analysis. HISAT2^2^ software was used to align the clean reads to the reference genome, generating mapped reads for further analysis.

**Differential expression analysis for validation**

To perform differential gene analysis based on the count matrix, we used the R package DESeq2 (v1.30.0)^3^. Additionally, since some samples had low quality when initially submitted, we re-submitted the samples to ensure their quality, and imported batch correction during the DESeq2 differential gene calculation.

Based on DESeq2, we obtained the foldchanges and P values for each transcript (with ENSEMBL as the id) after the interventions of different NP. The ENSEMBL id for each transcript was converted to ENTREZ id and gene symbol by function bitr() in R package clusterProfiler (v3.18.0)^4^. We calculated the accuracy of the model's predictions based on the following formula:

$$Acc_{class_{i}}=\frac{\left| intersection\left( {predict}_{class_{i}},truth_{class_{i}} \right) \right|}{\left| {predict}_{class_{i}} \right|}, i\in\{0,1\}$$

Here, $predict_{class_{i}}$ represented the genes predicted and classified by SETComp into $class_{i}$ with the softmax of the output no less than the defined threshold and $truth_{class_{i}}$ refers to all genes or differentially expressed genes belonging to $class_{i}$ determined by foldchanges. We performed the statistical analysis separately for both scenarios under the conditions of each NP intervention in each cell line.

Additionally, for the predictions classified as class 2, which indicates no significant intervention effect, we performed statistical analysis using the following formula:

$$Acc_{class_{2}}=\frac{\left| intersection\left( {predict}_{class_{2}},ns \right) \right|}{\left| {predict}_{class_{2}} \right|}$$

In which, $ns$ represented all the genes with P value no less than 0.05 and ${predict}_{class_{2}}$ has two calculation methods: one is for genes predicted to belong to $class_{2}$, and the other is for genes predicted not to belong to $class_{0}$or $class_{1}$.

**Supplementary Figures**

Supplementary Figure S1. Schematic diagram of the study design. This study pre-trained on large-scale compound–cell line transcriptomic data and fine-tuned on natural products–cell line transcriptomic data to predict transcriptomic changes induced by compounds or natural products across different cell lines.


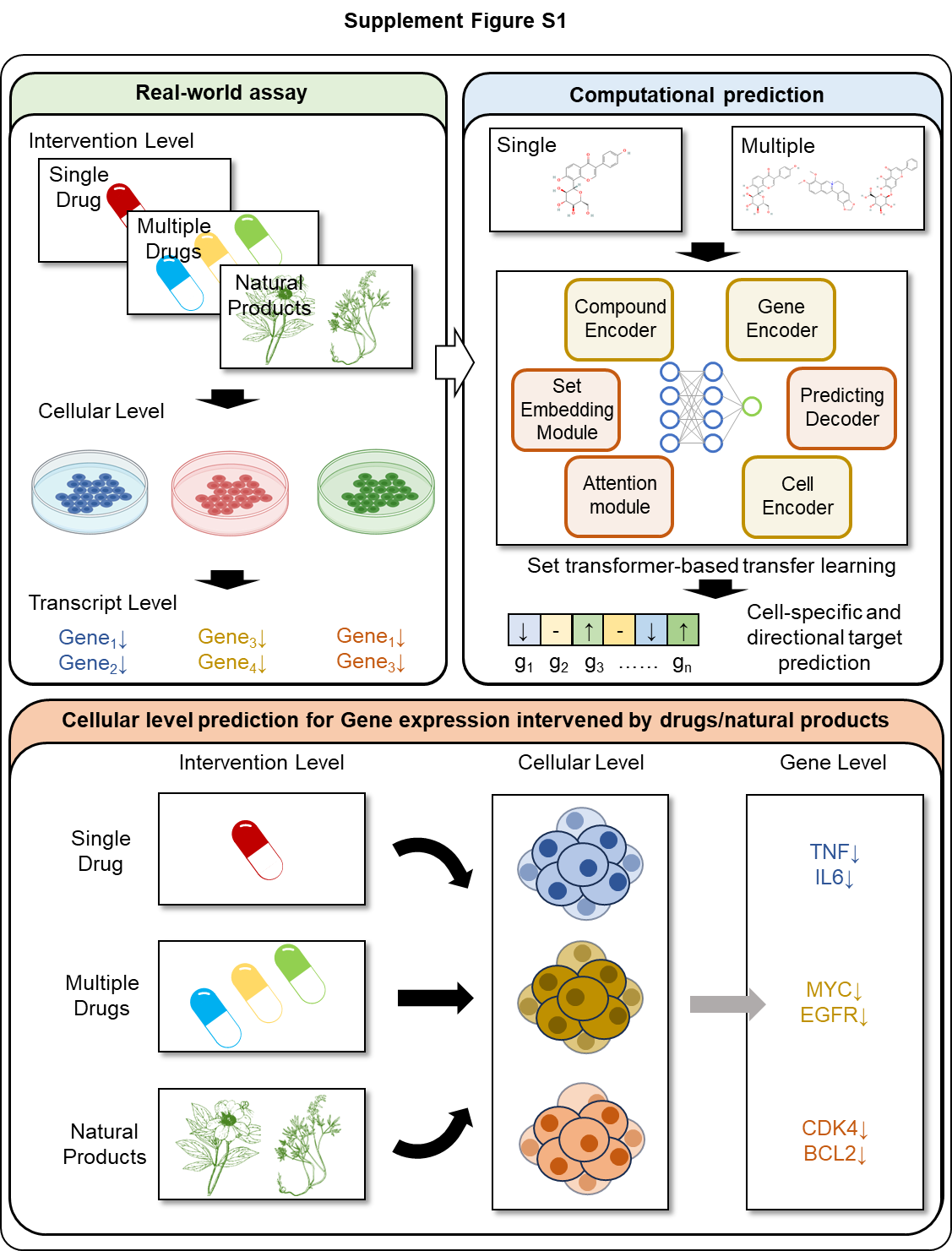


Supplementary Figure S2. Overview of the data involved in the study. (a) Summary and sources of the study data. (b) Histogram of the number of compound–cell line transcriptomic experiments. (c) Top 10 cell lines ranked by the number of compound–cell line experiments, along with the corresponding number of compounds involved. (d) UMAP dimensionality reduction of transcriptomes from untreated cell lines.


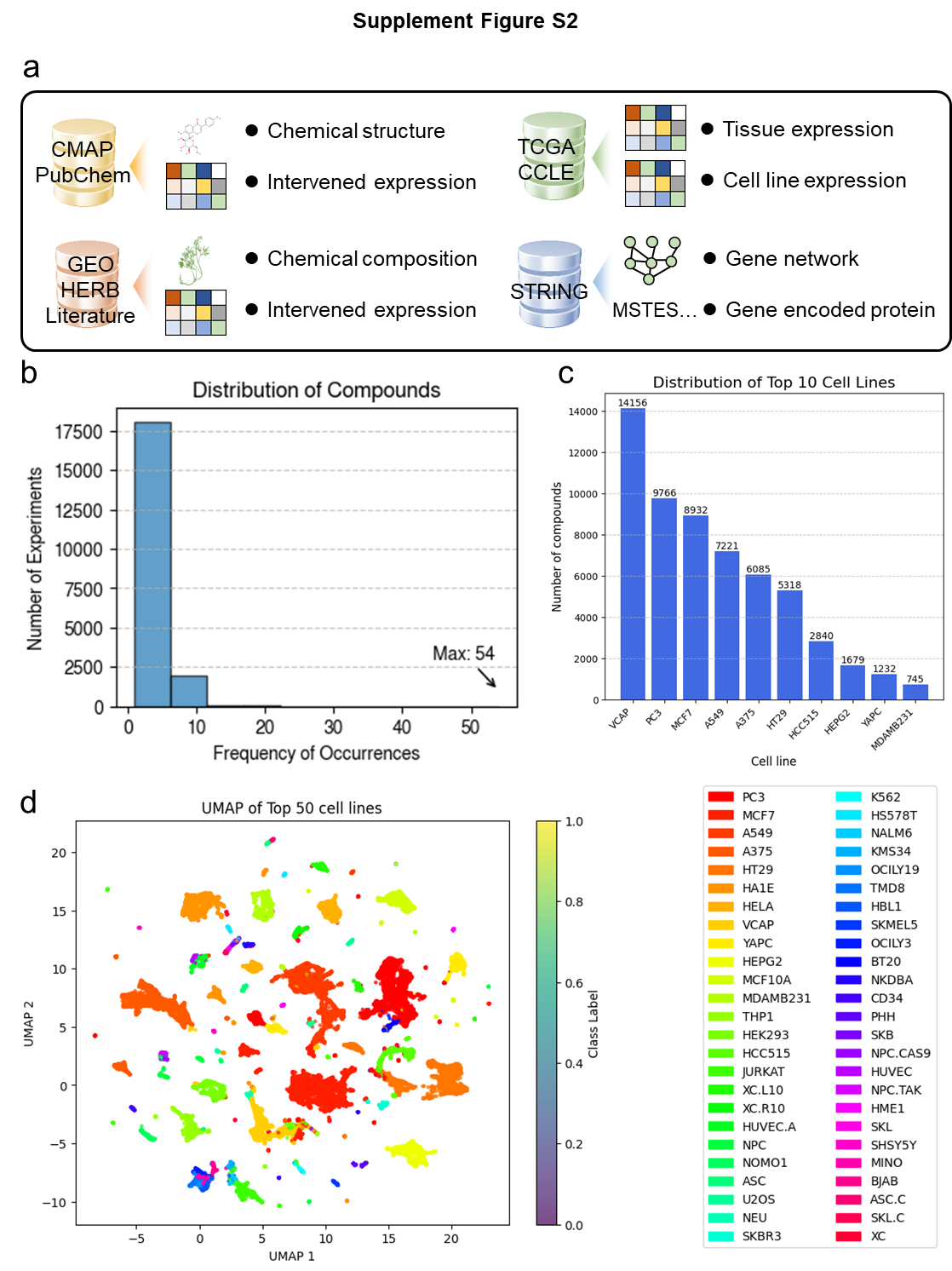


Supplementary Figure S3. Schematic diagram of the Set Embedding Module. The Set Embedding Module combines the Deep Sets model and the Set Transformer, forming different versions of SETComp based on the final feature aggregation method.


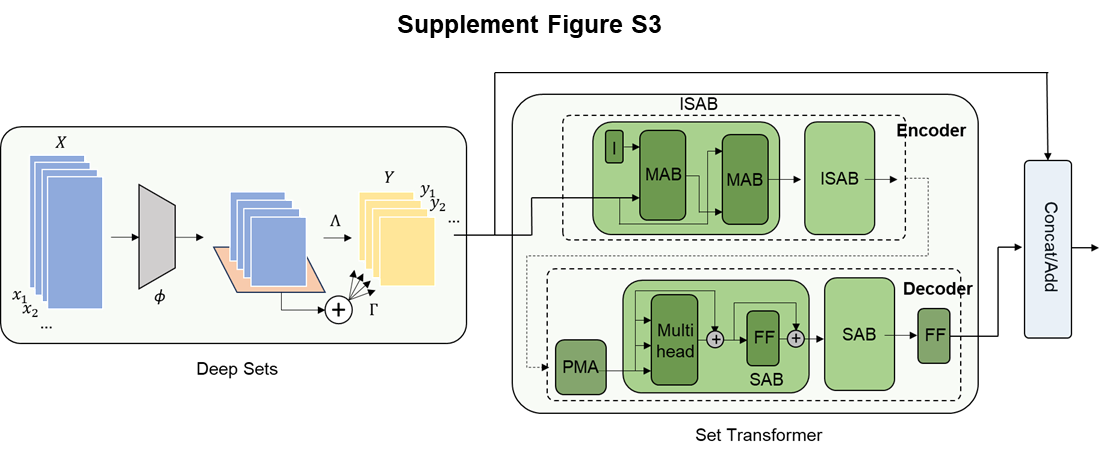


Supplementary Figure S4. Model performance and comparison in pre-training procedure. (a) ROC curves and AUC scores of different models on the test set. (b) Precision-Recall (PR) curves and AUPR scores of different models on the test set. (c) ROC curves and AUC scores for different classes of different models on the test set. (d) Comparison of Accuracy, F1 score, AUC, and AUPR of different models on the test set.


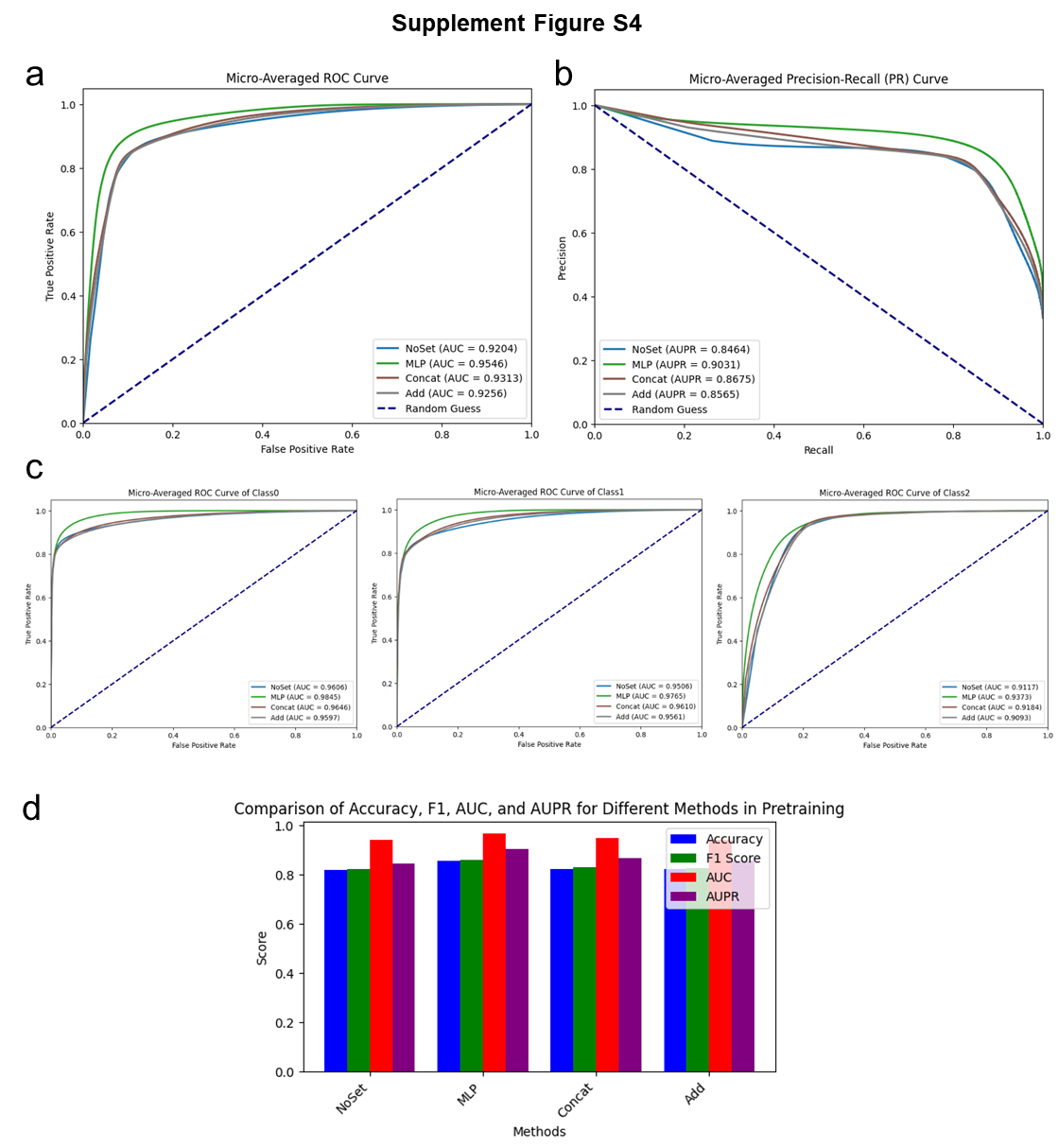


Supplementary Figure S5. ROC and PR curves of the Concat version and Add version of SETComp on classification tasks for different categories in the test set.


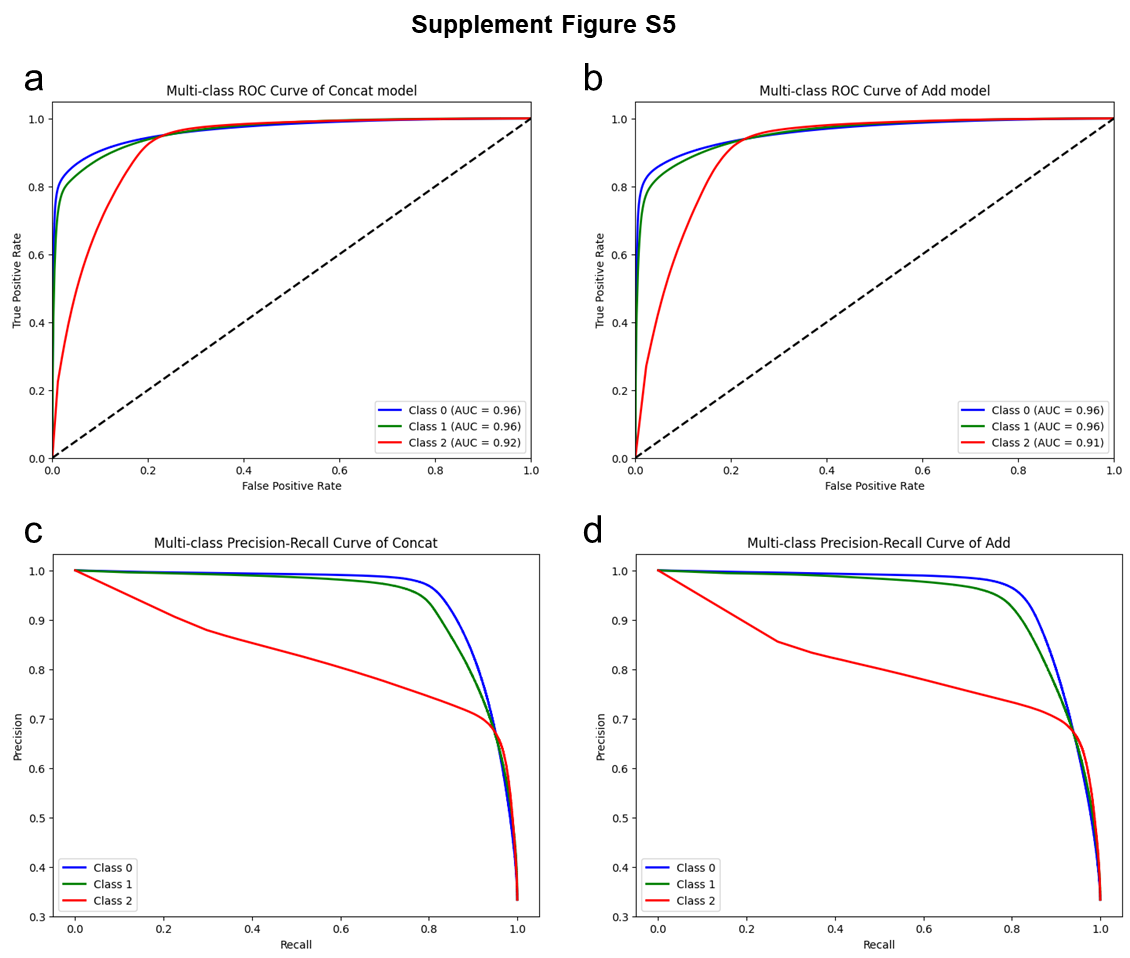


Supplementary Figure S6. Model performance and comparison of models pretrained on a small-scale (1/100) training set in pre-training procedure. (a) ROC curves and AUC scores of different models on the test set. (b) Precision-Recall (PR) curves and AUPR scores of different models on the test set. (c) ROC curves and AUC scores for different classes of different models on the test set. (d) Comparison of Accuracy, F1 score, AUC, and AUPR of different models on the test set.


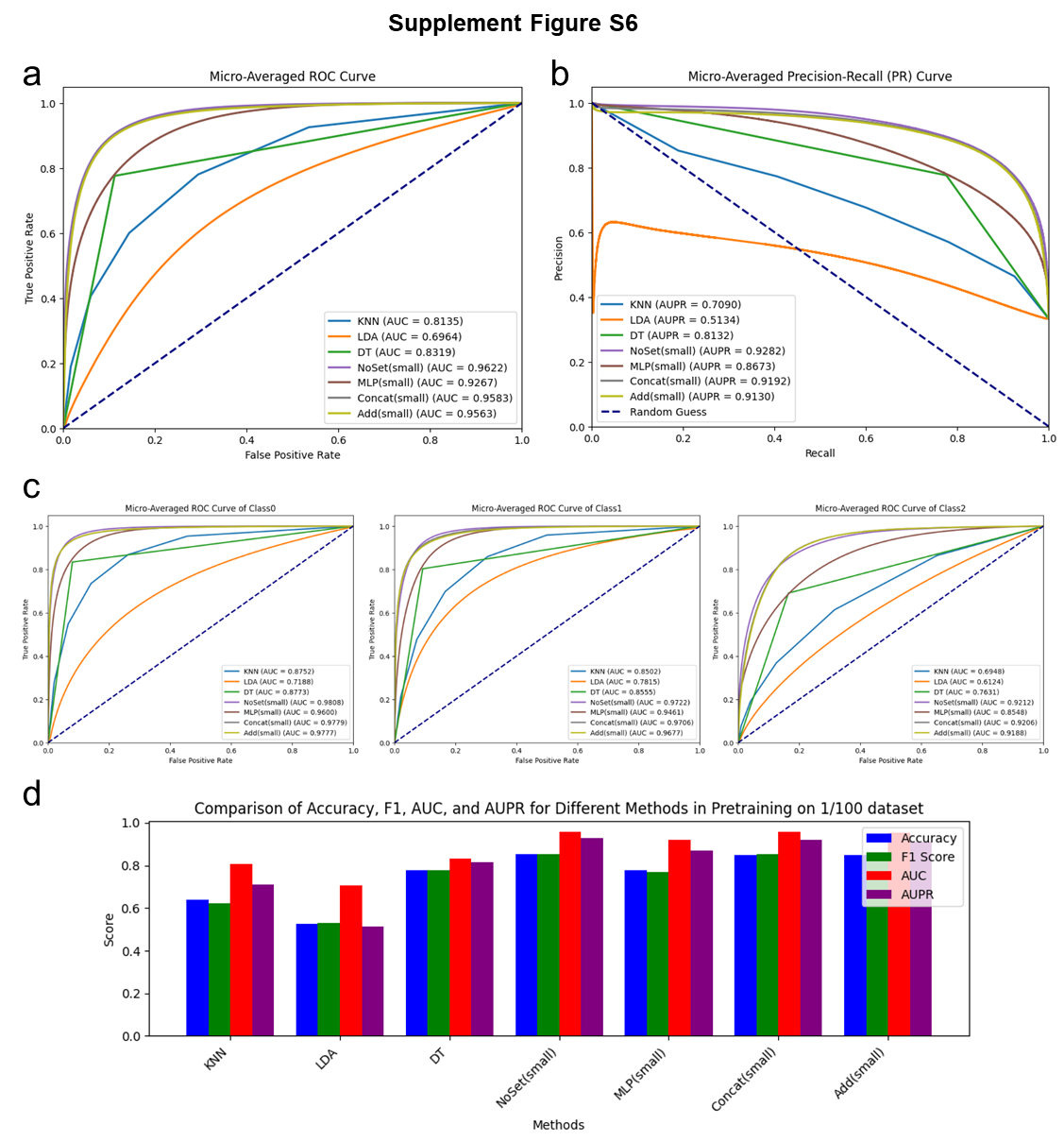


Supplementary Figure S7. Overview of the fine-tuning dataset. (a) Histogram of the number of compounds contained in different natural products. (b) Distribution of normalized total expression levels in transcriptomes after natural product intervention. (c) Correlation heatmap of transcriptomes following natural product intervention. (d) Correlation analysis between the number of compounds and expression levels in transcriptomes after natural product intervention.


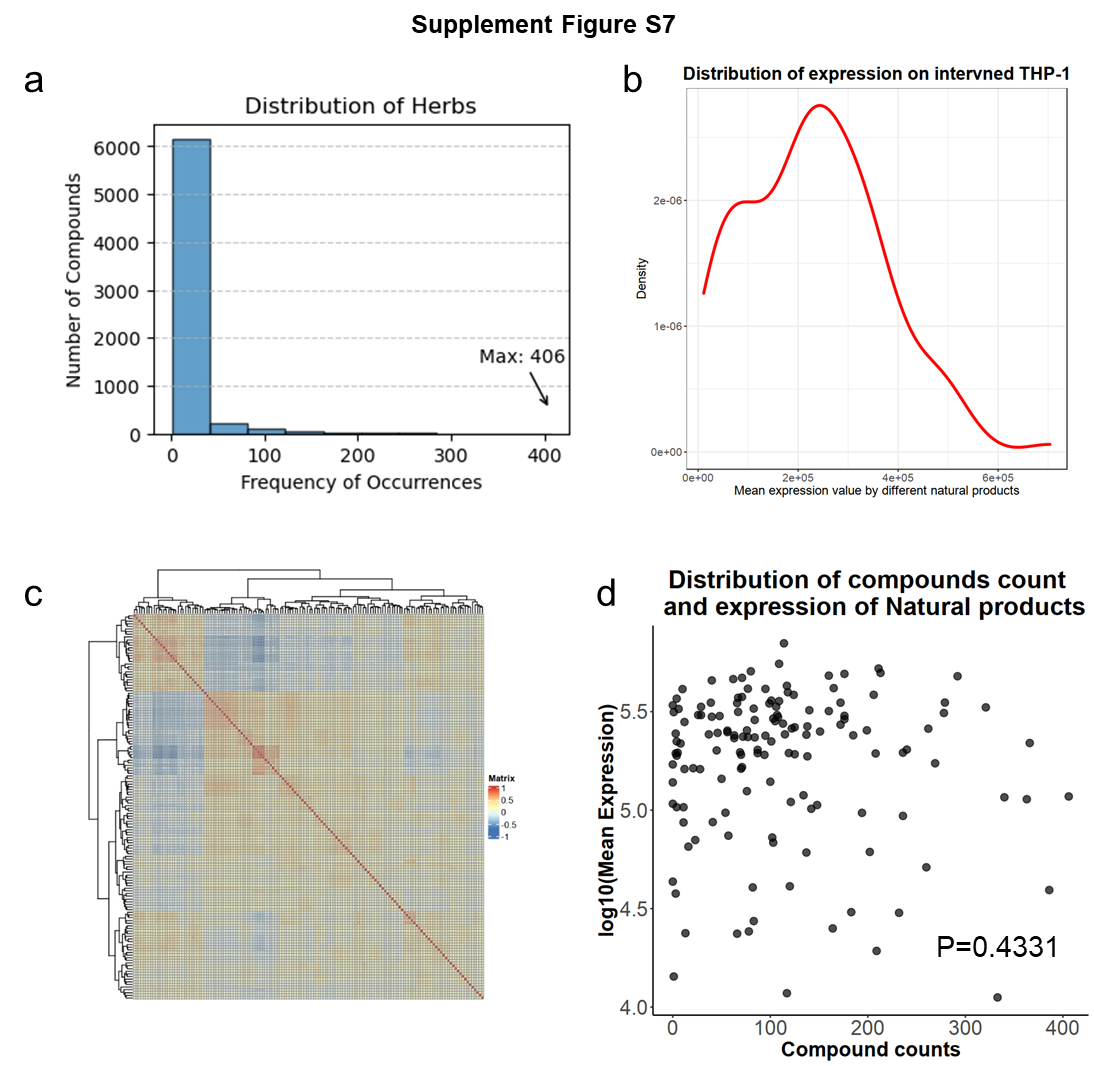


Supplementary Figure S8. Model performance and comparison of models in fine-tuning procedure. (a) Precision-Recall (PR) curves and AUPR scores of different models on the test set. (b) ROC curves and AUC scores for different classes of different models on the test set.


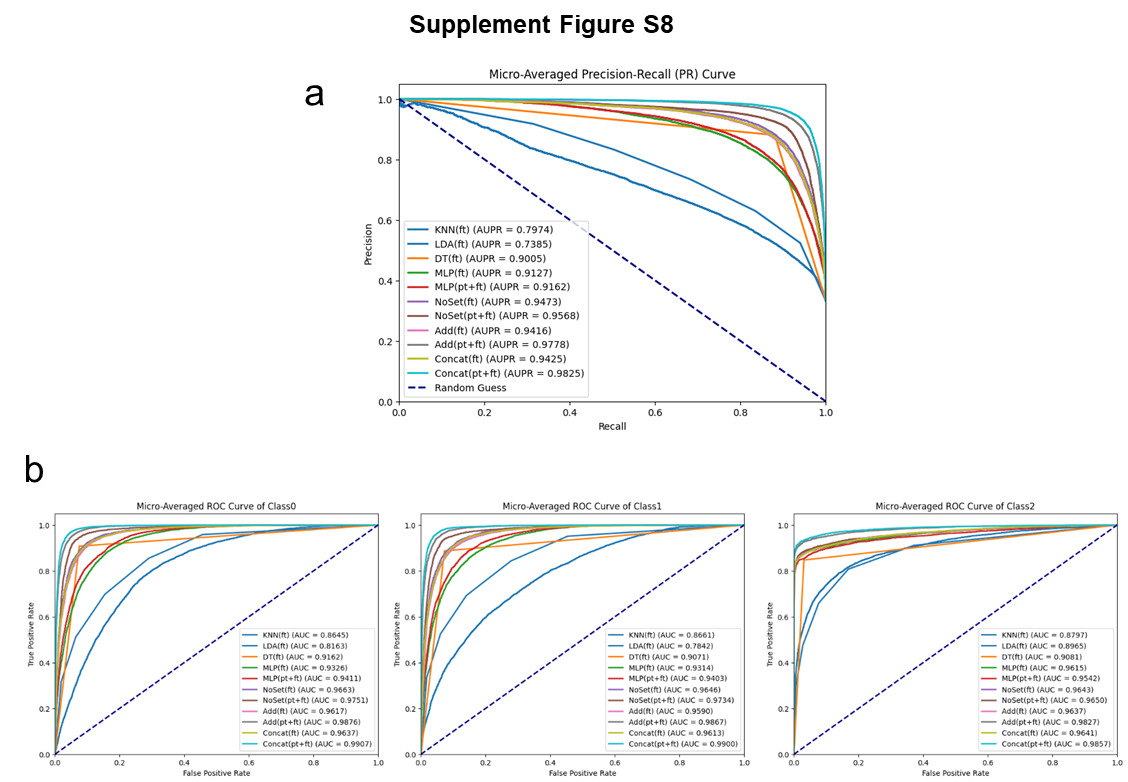


Supplementary Figure S9. Ablation Study 1: Model performance and comparison of pretrained and non-pretrained models in pre-training procedure. (a) ROC curves and AUC scores of different models on the test set. (b) Precision-Recall (PR) curves and AUPR scores of different models on the test set. (c) ROC curves and AUC scores for different classes of different models on the test set.


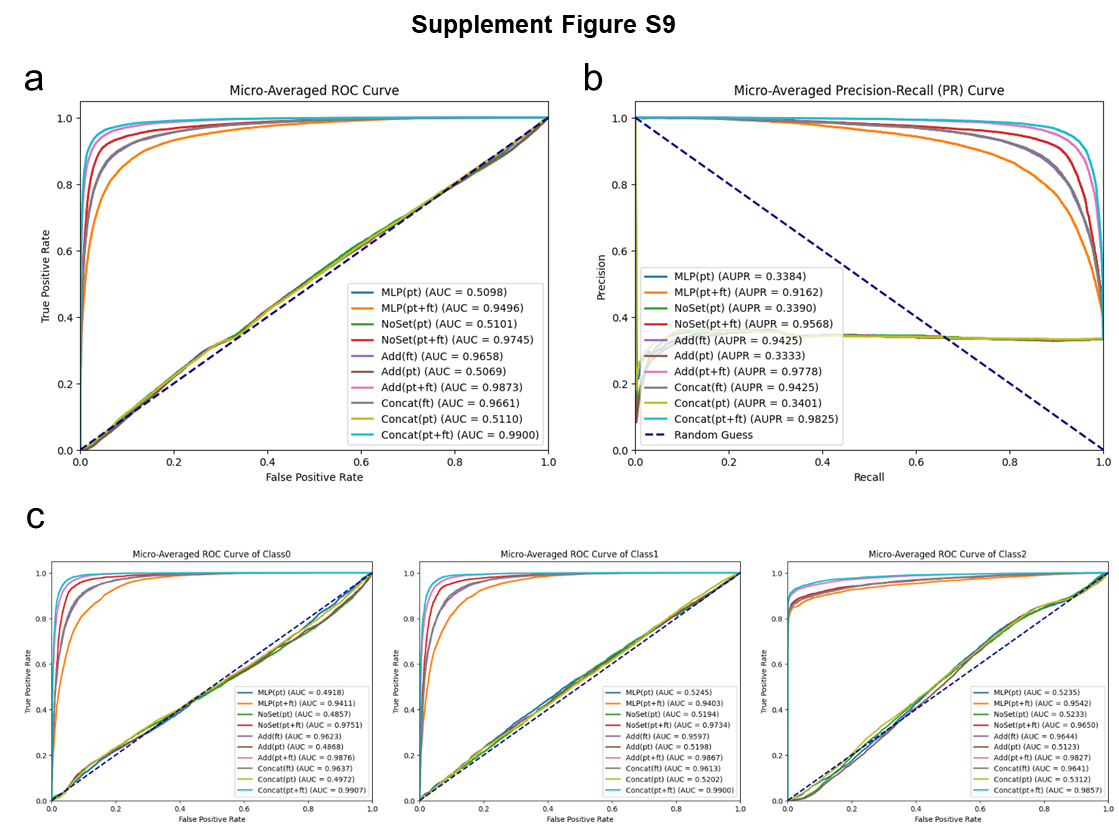


Supplementary Figure S10. Ablation Study 2: Analysis of the relationship between fine-tuning dataset size and model performance. (a) Accuracy of the Concat version at different fine-tuning dataset sizes. (b) F1 score of the Concat version at different fine-tuning dataset sizes. (c) Accuracy, F1 score, and AUC of the Add version at different fine-tuning dataset sizes.


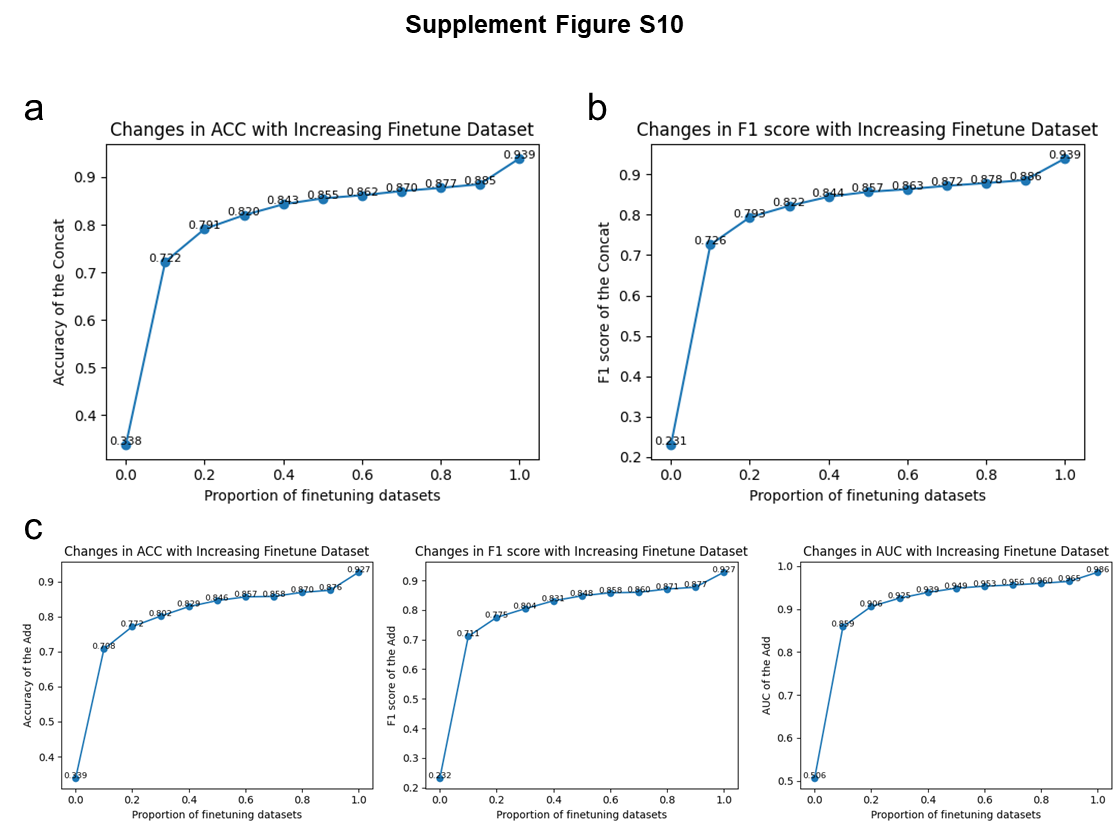


Supplementary Figure S11. Ablation Study 3: Impact of pretraining scale on model performance. (a) Validation loss curves of the Concat version model under different pretraining scales. (b) Validation loss curves of the Add version model under different pretraining scales. (c) Validation accuracy of the Add version model under different pretraining scales. (d) Validation AUC scores of the Add version model under different pretraining scales.


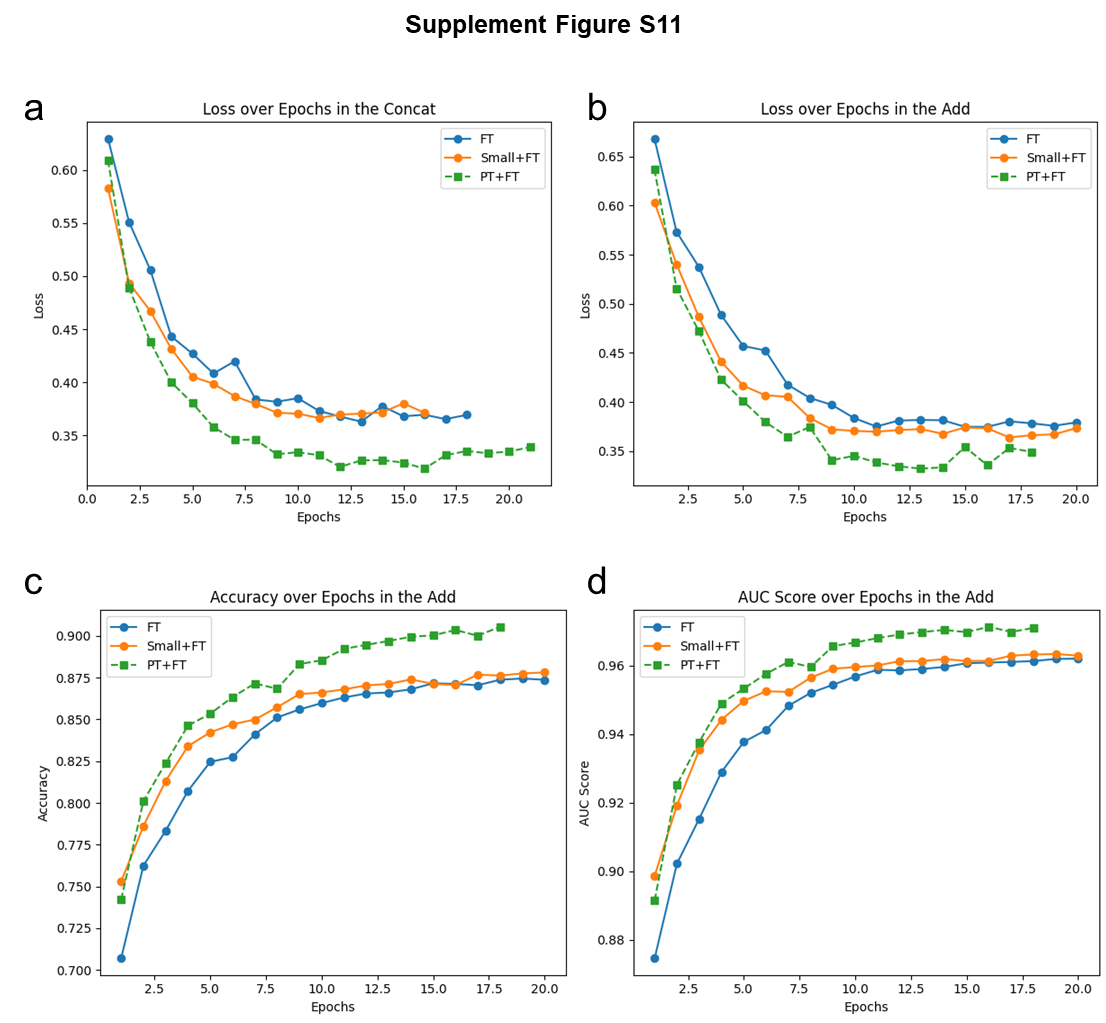


Supplementary Figure S12. Model performance and comparison of models in fine-tuning procedure (with baseline machine-learning models). (a) ROC curves and AUC scores of different models on the test set. (b) Precision-Recall (PR) curves and AUPR scores of different models on the test set. (c) ROC curves and AUC scores for different classes of different models on the test set. (d) Comparison of Accuracy, F1 score, AUC, and AUPR of different models on the test set.


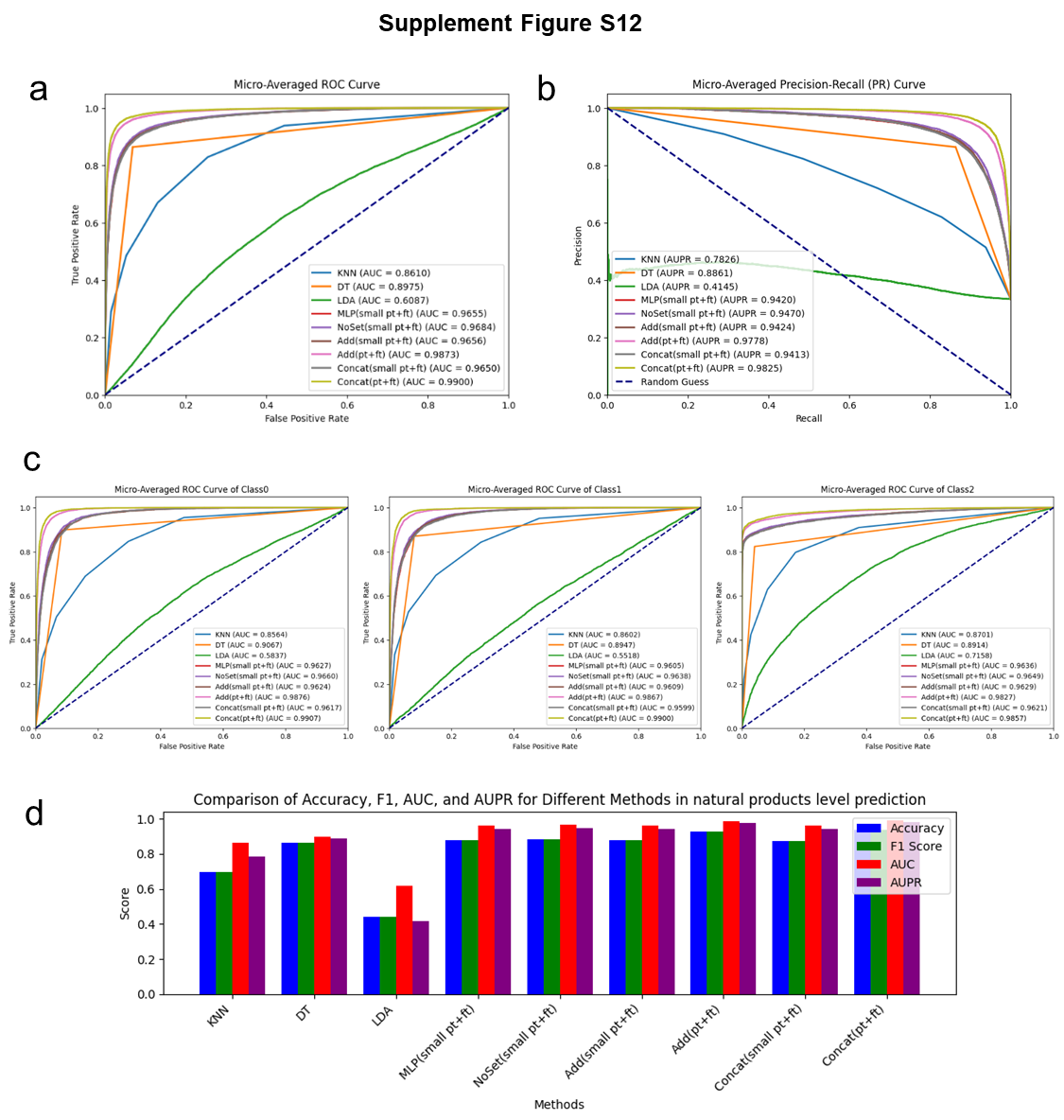


Supplementary Figure S13. Performance and comparison of the model on unseen natural products as the test set. (a) ROC curves and AUC scores of different models on the test set. (b) Precision-Recall (PR) curves and AUPR scores of different models on the test set. (c) ROC curves and AUC scores for different classes of different models on the test set.


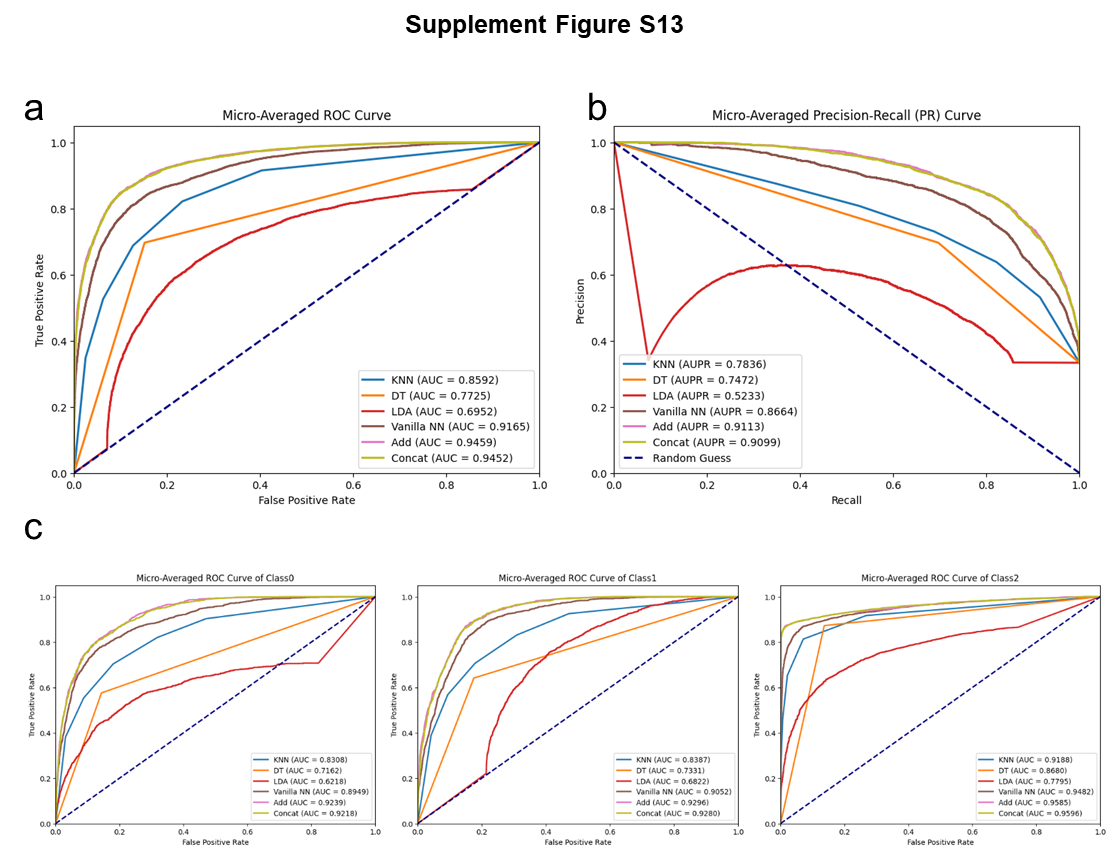


Supplementary Figure S14. Correlation between the model-predicted class softmax outputs and the true log fold change in transcriptomes for unseen natural products.


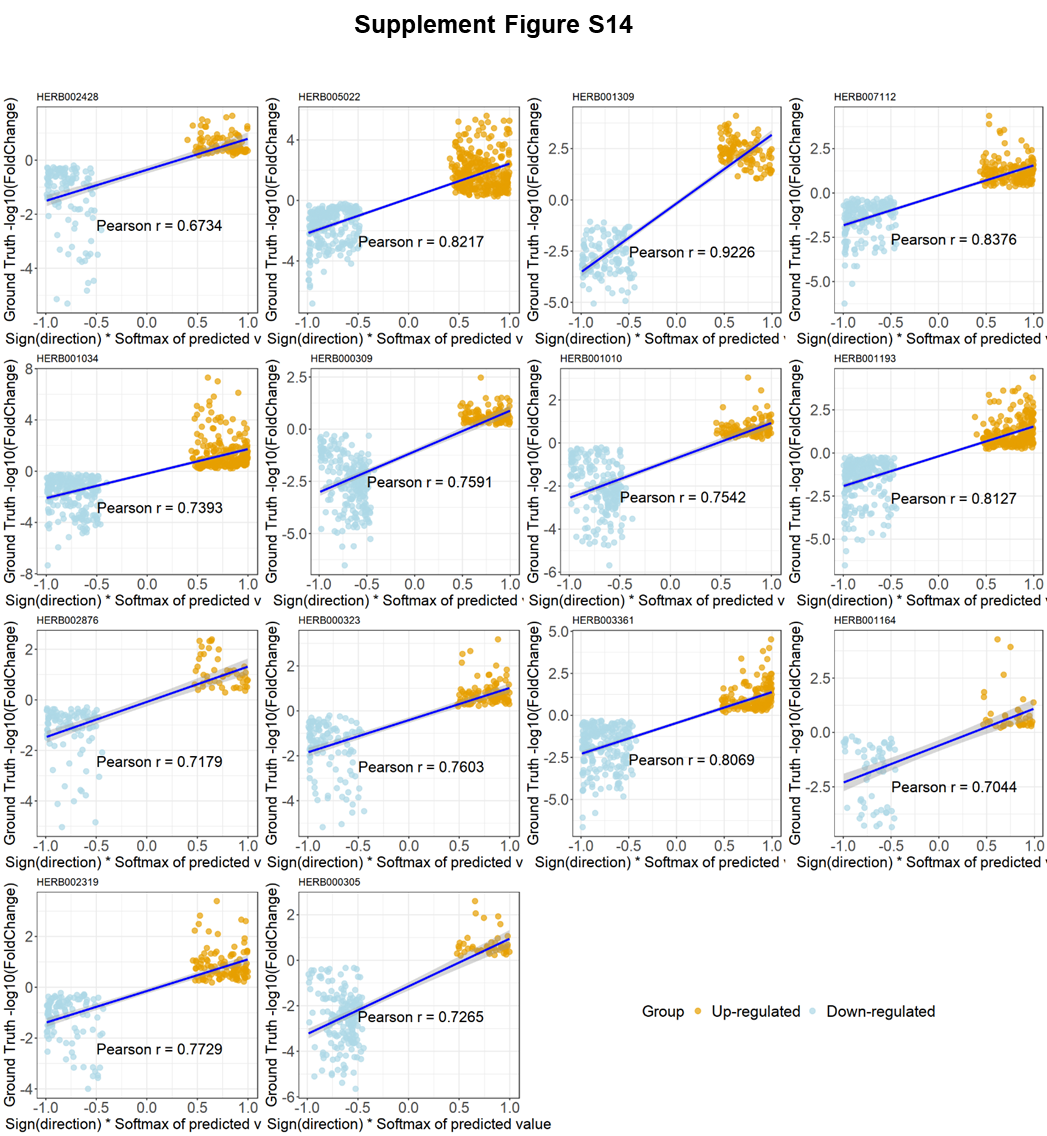


Supplementary Figure S15. Model performance validation on the A549 cell line. (a) Differentially expressed genes in A549 cells after intervention with different natural products. (b) Model performance for HERB004694 on the A549 cell line, evaluated on the whole genome and the differentially expressed genes. (c) Model performance for HERB001211 and HERB002560 on the A549 cell line, evaluated on the whole genome.


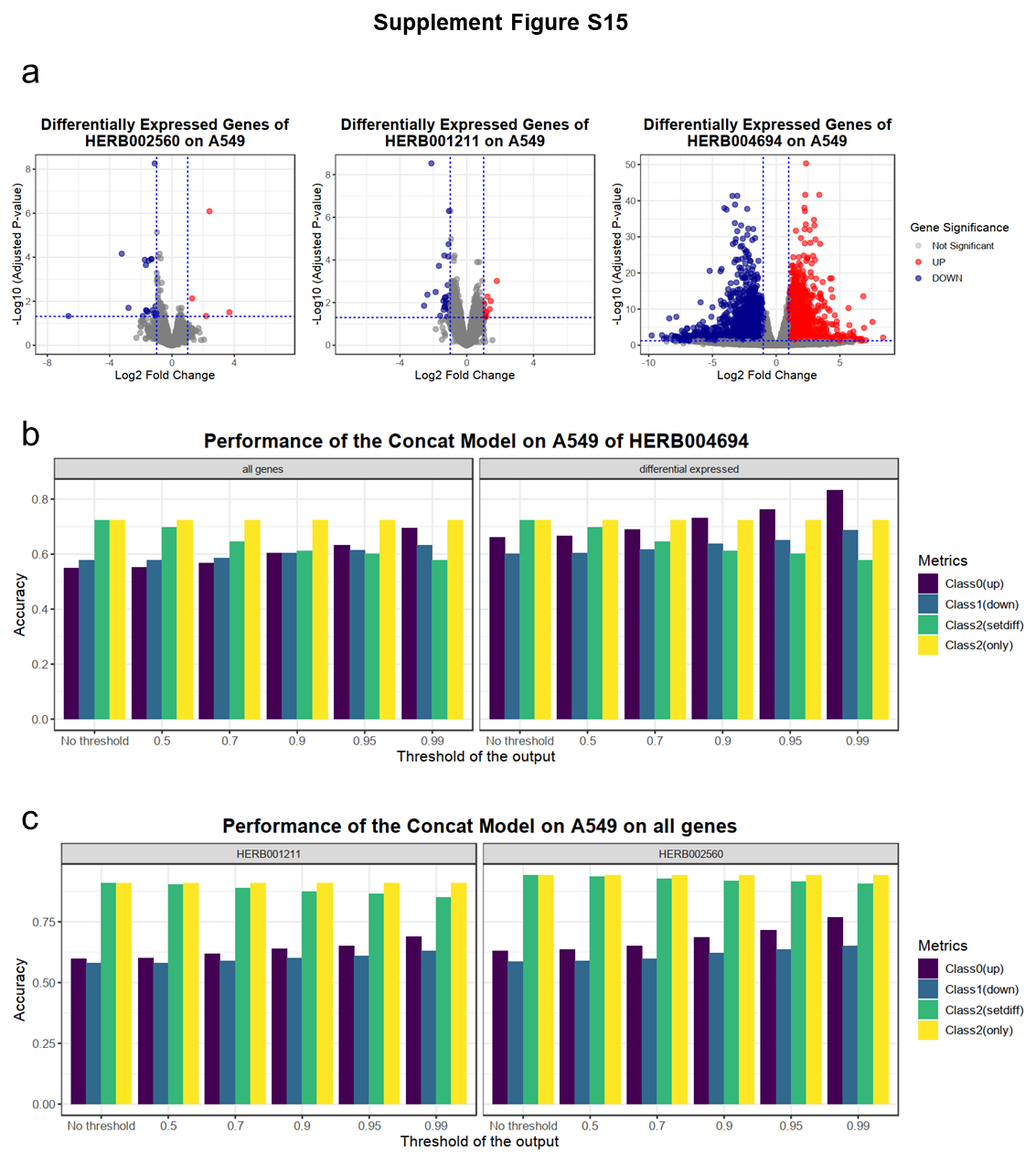


Supplementary Figure S16. Correlation between the model predictions and the actual experimental log2 fold change in the A549 cell line. (a) Correlation between the model-predicted class softmax outputs and the true experimental log2 fold change across the whole genome. (b) Correlation between the model-predicted class softmax outputs and the true experimental log2 fold change among differentially expressed genes after intervention with various natural products. (c) Correlation between the model-predicted class softmax outputs and the true experimental log2 fold change across the whole genome after intervention with various natural products.


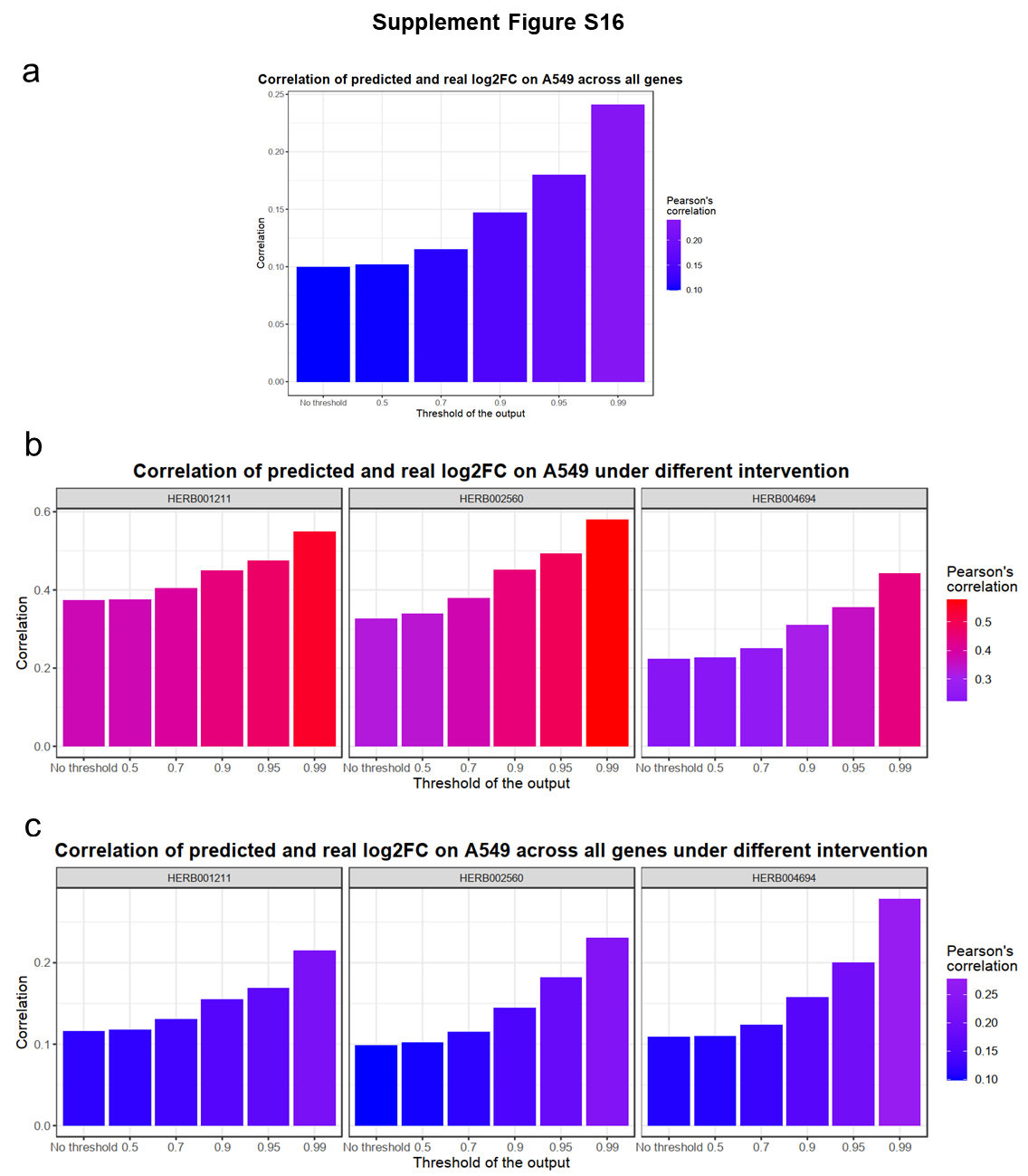


Supplementary Figure S17. Analysis of tumor-related genes in cell lines potentially intervened by the model. (a) Changes in genes with high model-predicted scores and significant differential expression in non-small cell lung cancer (NSCLC) TCGA data before and after intervention in the A549 cell line. (b) Differential expression of these high-scoring genes in the LUAD TCGA dataset. (c) Differential expression of these high-scoring genes in the LUSC TCGA dataset.


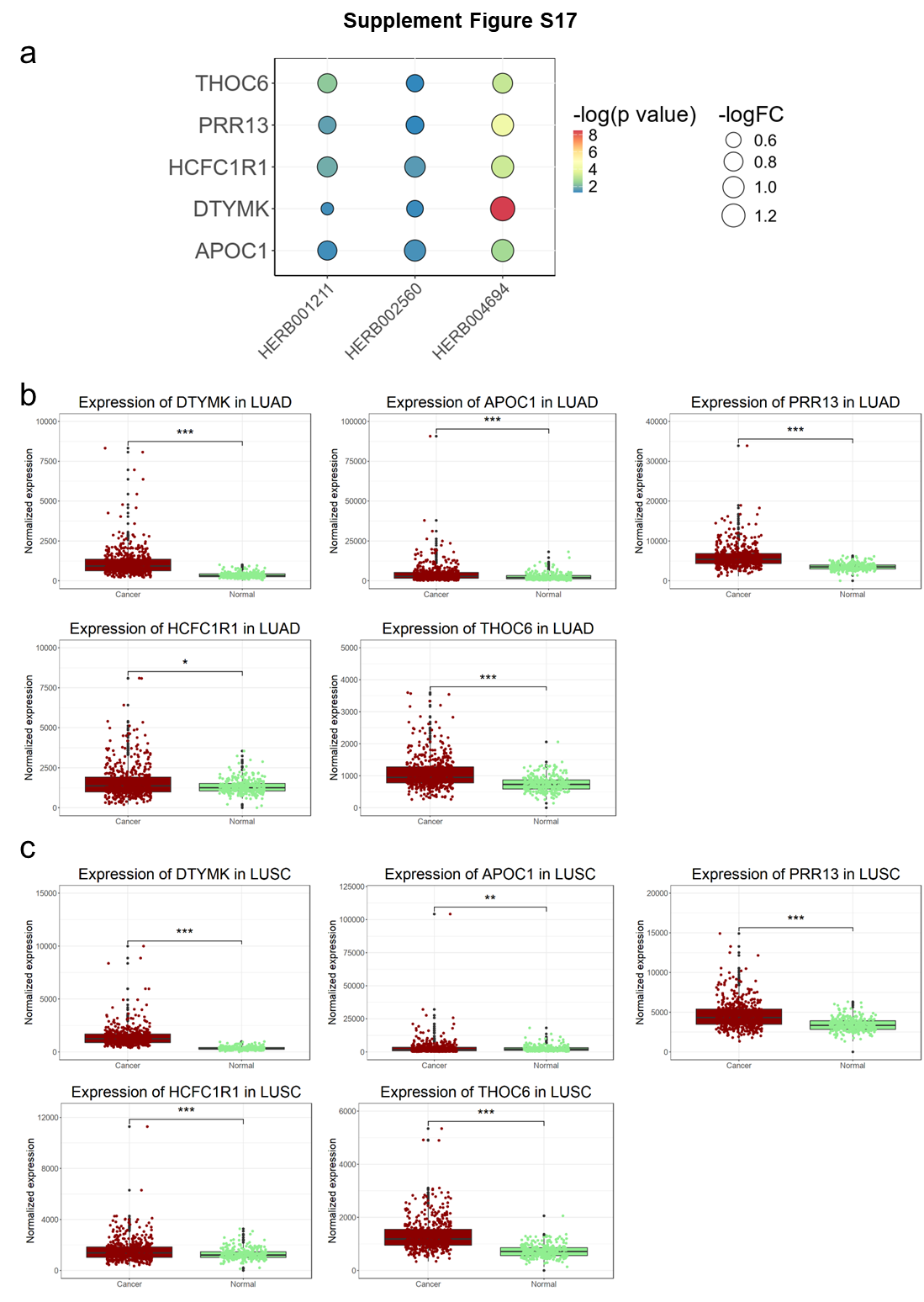


Supplementary Figure S18. Supplementary results 1 for the application of the model in mechanism analysis of complex systems such as natural products. (a) Intersection and union of genes associated with high scores in the predicted natural products. (b) Top-ranked genes in different score categories in MCF-7 and A549 after intervention with HERB001211 as predicted by the model. (c) Top-ranked genes in different score categories in MCF-7 and A549 after intervention with HERB002560 as predicted by the model. (d) Top-ranked genes in different score categories in MCF-7 and A549 after intervention with HERB004694 as predicted by the model.


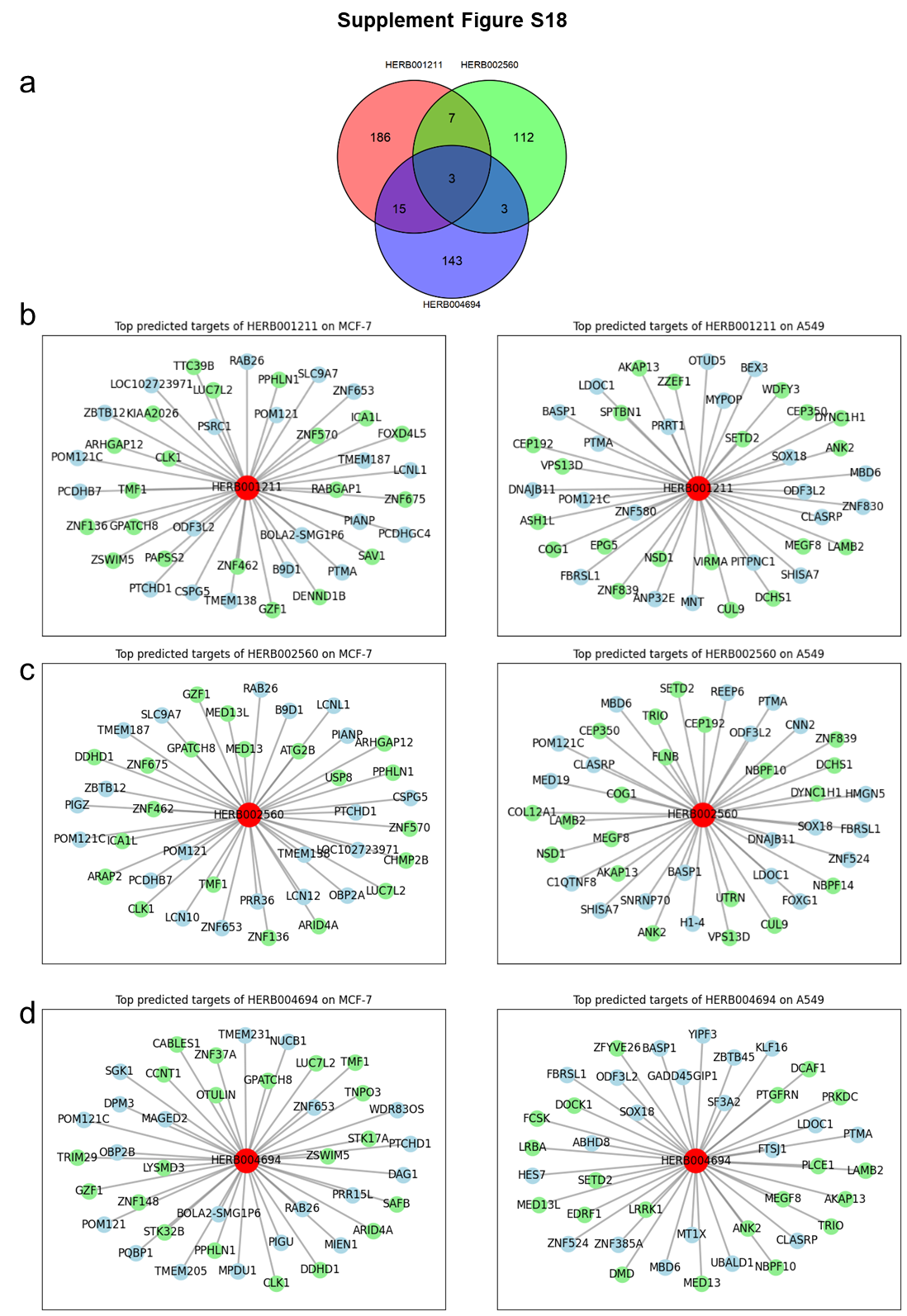


Supplementary Figure S19. Supplementary results 2 for the application of the model in mechanism analysis of complex systems such as natural products. (a) Mechanism uncovering of potential intervention pathways of HERB001211 in MCF-7 cell lines. (b) Mechanism uncovering of potential intervention pathways of HERB004694 in MCF-7 cell lines.


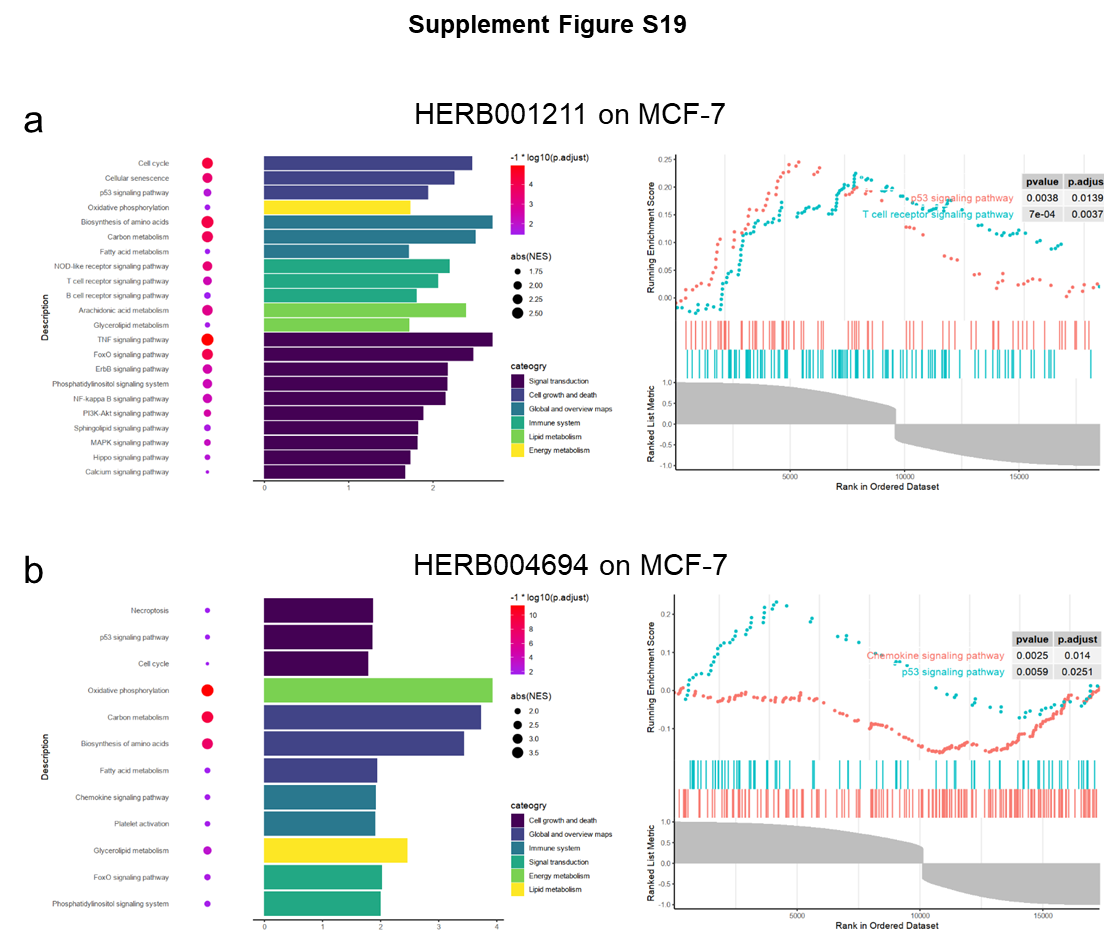


Supplementary Figure S20. Supplementary results 3 for the application of the model in mechanism analysis of complex systems such as natural products. (a) Mechanism uncovering of potential intervention pathways of HERB001211 in A549 cell lines. (b) Mechanism uncovering of potential intervention pathways of HERB004694 in A549 cell lines.


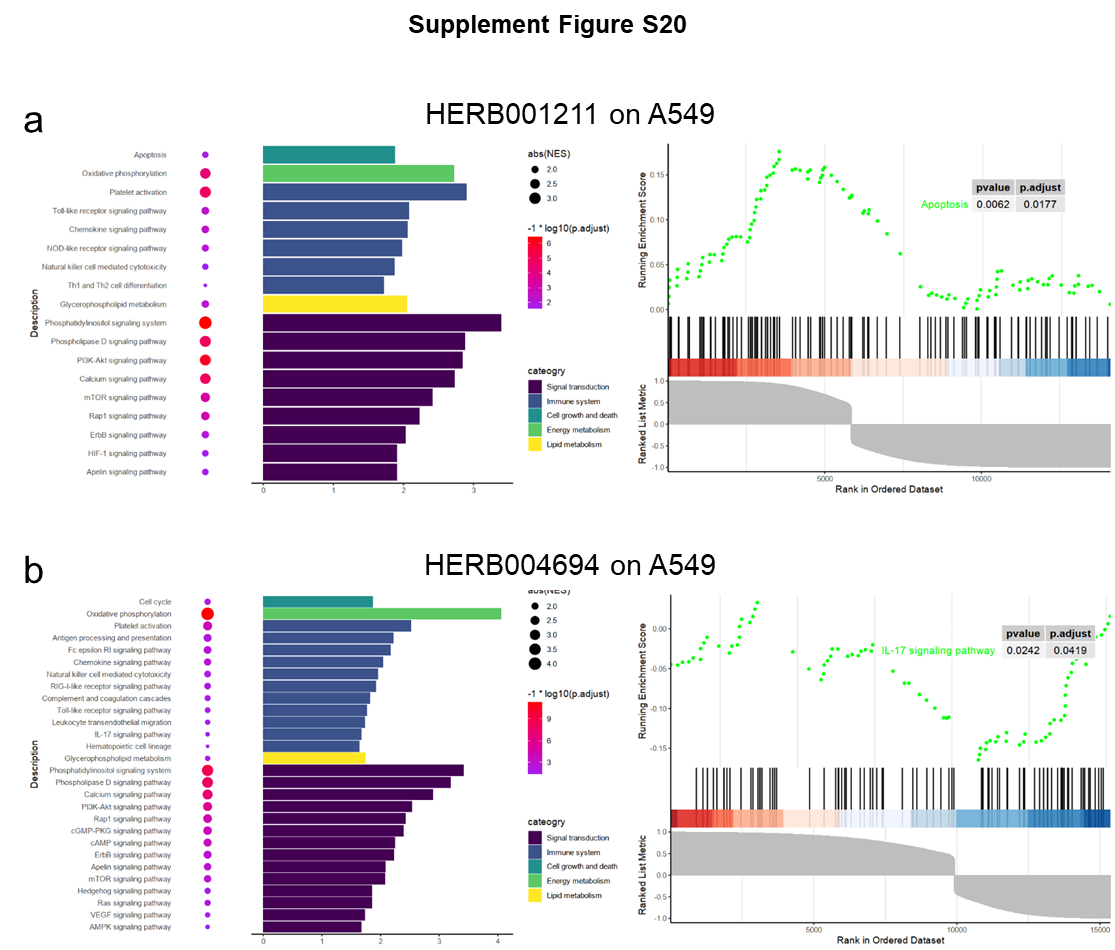


Supplementary Figure S21. Supplementary results 4 for the application of the model in mechanism analysis of complex systems such as natural products. (a) Mechanism uncovering of potential intervention pathways of HERB002560 in MCF-7 cell lines with 0.7 as the threshold. (b) Mechanism uncovering of potential intervention pathways of HERB002560 in A549 cell lines with 0.7 as the threshold. (c) Mechanism uncovering of potential intervention pathways of HERB002560 in MCF7 cell lines with 0.9 as the threshold. (d) Mechanism uncovering of potential intervention pathways of HERB002560 in A549 cell lines with 0.9 as the threshold.


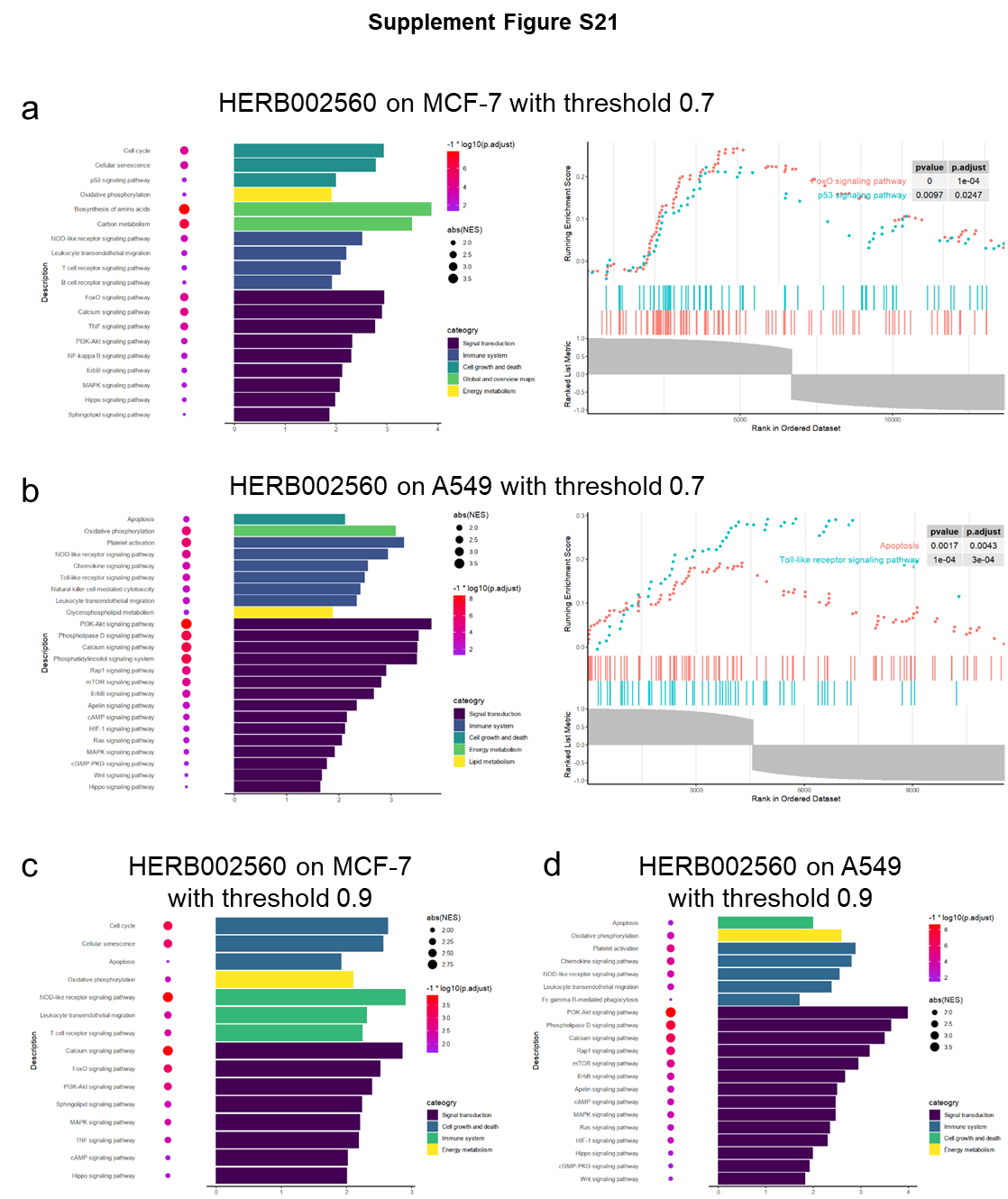


Supplementary Figure S22. Supplementary results of mechanism repositioning for complex systems such as natural products using the model. (a) Potentially intervened KEGG pathways of HERB004609. (b) Potentially intervened KEGG pathways of HERB005900. (c) Potentially intervened KEGG pathways of HERB002319. (d) Potentially intervened KEGG pathways of HERB004694. (e) Repositioning of HERB004694 based on SETComp predictions. (f) Repositioning of HERB004609 based on SETComp predictions.


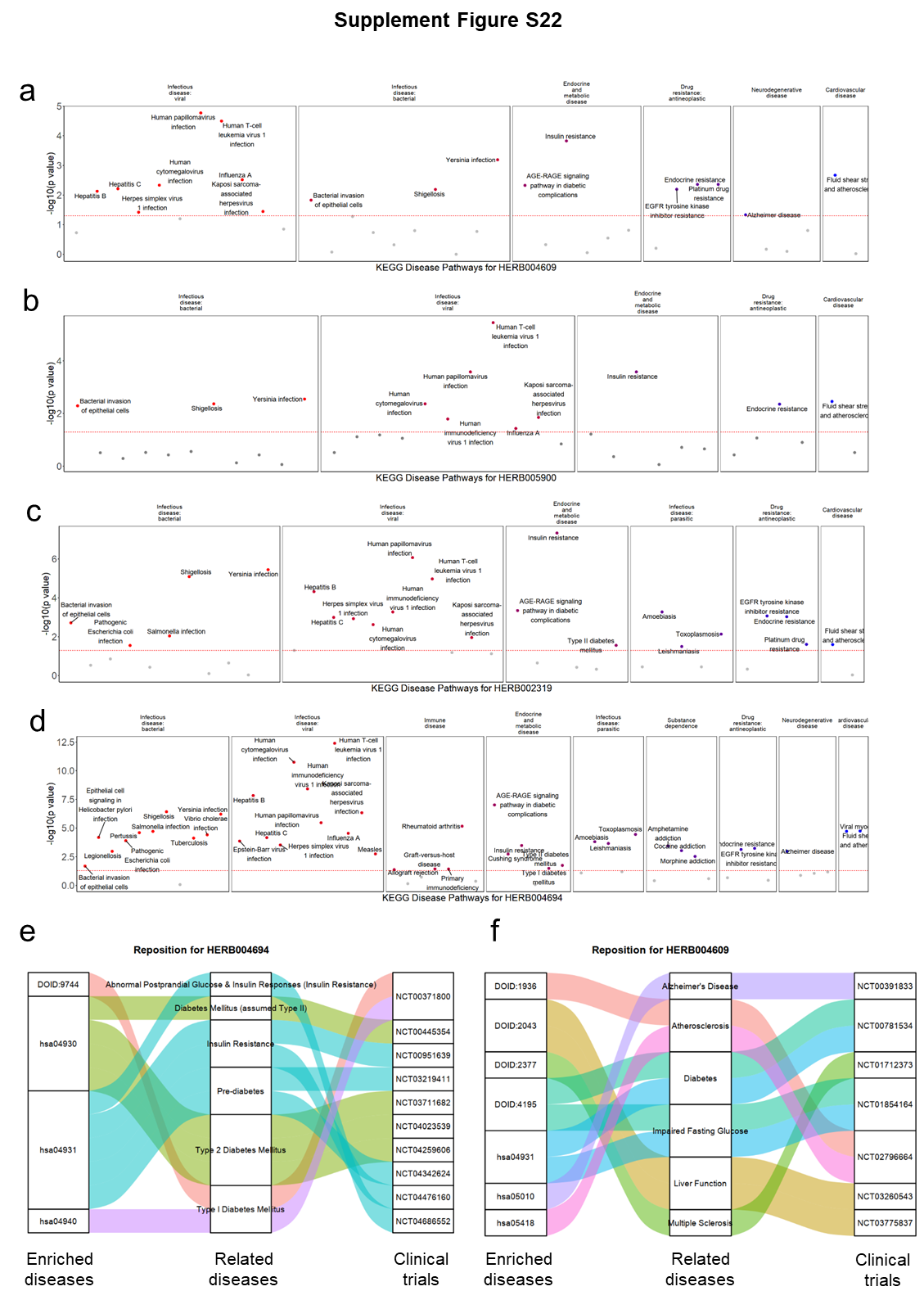


**Supplementary Tables**

Supplementary Table S1. Grid search parameters during pretraining of the Concat version

| **Parameters** | **Searching range** |
| --- | --- |
| batch size | 1024, 512, 256, 128, 64 |
| learning rate | 1e-3, 1e-4, 1e-5, 1e-6 |
| L2 regularization | 1e-5, 1e-4, 1e-3, 1e-2 |
| dropout | 0.1, 0.2, 0.5, 0.8 |
| dimension in MLP layer | 512, 1024 |
| dimension in Set Transformer layer | 512, 1024, 2048 |

Supplementary Table S2. Grid search parameters during pretraining of the Add version

| **Parameters** | **Searching range** |
| --- | --- |
| batch size | 1024, 512, 256, 128, 64 |
| learning rate | 1e-3, 1e-4, 1e-5, 1e-6 |
| L2 regularization | 1e-5, 1e-4, 1e-3, 1e-2 |
| dropout | 0.1, 0.2, 0.5, 0.8 |

Supplementary Table S3. Training performance of the top 10 epochs with the best parameters in grid search of the Concat version

| **epoch** | **train_loss** | **val_loss** | **val_acc** | **val_auc** |
| --- | --- | --- | --- | --- |
| 0 | 0.10441394 | 0.05324341 | 0.9812739 | 0.9987965 |
| 1 | 0.05155225 | 0.03958947 | 0.986271 | 0.9993037 |
| 2 | 0.04112046 | 0.03384728 | 0.9883974 | 0.9994766 |
| 3 | 0.03519382 | 0.02904077 | 0.9901183 | 0.9996113 |
| 4 | 0.03123626 | 0.02632332 | 0.9910326 | 0.999678 |
| 5 | 0.02828881 | 0.02437879 | 0.9917747 | 0.9997238 |
| 6 | 0.02604143 | 0.02251237 | 0.992409 | 0.9997612 |
| 7 | 0.02424367 | 0.02061381 | 0.9929859 | 0.9998 |
| 8 | 0.02271379 | 0.01918993 | 0.9934423 | 0.9998263 |
| 9 | 0.02131978 | 0.01939688 | 0.9935078 | 0.9998265 |

Supplementary Table S4. Training performance of the top 10 epochs with the best parameters in grid search of the Add version

| **epoch** | **train_loss** | **val_loss** | **val_acc** | **val_auc** |
| --- | --- | --- | --- | --- |
| 0 | 0.10701013 | 0.0540627 | 0.9811048 | 0.9987419 |
| 1 | 0.0520684 | 0.03944144 | 0.9862926 | 0.9993043 |
| 2 | 0.04086678 | 0.03285723 | 0.9886795 | 0.9995105 |
| 3 | 0.03480242 | 0.02901058 | 0.9901946 | 0.9996068 |
| 4 | 0.03088879 | 0.02569392 | 0.9912389 | 0.9996935 |
| 5 | 0.02794328 | 0.02301959 | 0.9921398 | 0.9997486 |
| 6 | 0.02564236 | 0.02184432 | 0.9925949 | 0.999774 |
| 7 | 0.02374276 | 0.02015439 | 0.9931734 | 0.9998074 |
| 8 | 0.02217382 | 0.01901249 | 0.9935783 | 0.999829 |
| 9 | 0.02084753 | 0.01827641 | 0.9938413 | 0.999837 |

Supplementary Table S5. Model parameter scale and performance during pretraining

| **Methods** | **Parameters** | **Accuracy** | **F1 Score** | **AUC** |
| --- | --- | --- | --- | --- |
| NoSet | 96.11M | 0.8177 | 0.8228 | 0.9410 |
| MLP | 5.07M | **0.8581** | **0.8611** | **0.9661** |
| Concat | 199.75M | 0.8250 | 0.8299 | 0.9480 |
| Add | 173.01M | 0.8216 | 0.8266 | 0.9417 |

Supplementary Table S6. Performance and comparison of models pretrained on a small-scale training set

| **Methods** | **Accuracy** | **F1 Score** | **AUC** |
| --- | --- | --- | --- |
| KNN | 0.6401 | 0.6237 | 0.8067 |
| LDA | 0.5258 | 0.5297 | 0.7042 |
| DT | 0.7759 | 0.7763 | 0.8319 |
| NoSet(small) | **0.8517** | **0.8504** | **0.9581** |
| MLP(small) | 0.7769 | 0.7684 | 0.9203 |
| Concat(small) | 0.8495 | 0.8509 | 0.9564 |
| Add(small) | 0.8479 | 0.8495 | 0.9547 |

Supplementary Table S7. Grid search parameters during fine-tuning of the Concat version

| **Parameters** | **Searching range** |
| --- | --- |
| batch size | 256, 128, 64 |
| learning rate | 1e-3, 1e-4, 1e-5, 1e-6 |
| L2 regularization | 1e-5, 1e-4, 1e-3, 1e-2 |
| dropout | 0.1, 0.2, 0.5, 0.8 |

Supplementary Table S8. Model performance and comparison during fine-tuning

| **Methods** | **Accuracy** | **F1 Score** | **AUC** |
| --- | --- | --- | --- |
| KNN(ft) | 0.7088 | 0.7097 | 0.8701 |
| LDA(ft) | 0.6627 | 0.6620 | 0.8325 |
| DT(ft) | 0.8804 | 0.8806 | 0.9105 |
| MLP(ft) | 0.8290 | 0.8316 | 0.9419 |
| MLP(pt+ft) | 0.8376 | 0.8399 | 0.9452 |
| NoSet(ft) | 0.8812 | 0.8821 | 0.9651 |
| NoSet(pt+ft) | 0.9065 | 0.9067 | 0.9711 |
| Add(ft) | 0.8737 | 0.8748 | 0.9615 |
| Add(pt+ft) | 0.9270 | 0.9272 | 0.9856 |
| Concat(ft) | 0.8758 | 0.8768 | 0.9630 |
| Concat(pt+ft) | **0.9386** | **0.9387** | **0.9888** |

Supplementary Table S9. Ablation Study 1: Performance and comparison of pretrained versus non-pretrained models during fine-tuning

| **Methods** | **Accuracy** | **F1 Score** | **AUC** |
| --- | --- | --- | --- |
| Add(ft) | 0.8737 | 0.8748 | 0.9615 |
| Add(pt) | 0.3386 | 0.2323 | 0.5063 |
| Add(pt+ft) | **0.9270** | **0.9272** | **0.9856** |
| Concat(ft) | 0.8758 | 0.8768 | 0.9630 |
| Concat(pt) | 0.3379 | 0.2308 | 0.5162 |
| Concat(pt+ft) | **0.9386** | **0.9387** | **0.9888** |

Supplementary Table S10. Ablation Study 2: Performance and comparison of models pretrained at different scales during fine-tuning

| **Methods** | **Accuracy** | **F1 Score** | **AUC** |
| --- | --- | --- | --- |
| KNN | 0.6945 | 0.6948 | 0.8623 |
| DT | 0.8633 | 0.8633 | 0.8976 |
| LDA | 0.4393 | 0.4412 | 0.6174 |
| MLP(small pt+ft) | 0.8766 | 0.8778 | 0.9623 |
| NoSet(small pt+ft) | 0.8818 | 0.8827 | 0.9649 |
| Add(small pt+ft) | 0.8781 | 0.8791 | 0.9621 |
| Add(pt+ft) | 0.9270 | 0.9272 | 0.9856 |
| Concat(small pt+ft) | 0.8737 | 0.8748 | 0.9612 |
| Concat(pt+ft) | **0.9386** | **0.9387** | **0.9888** |

Supplementary Table S11. Performance and comparison of the model on unseen natural products

| **Methods** | **Accuracy** | **F1 Score** | **AUC** |
| --- | --- | --- | --- |
| KNN | 0.7062 | 0.7108 | 0.8628 |
| DT | 0.6966 | 0.6919 | 0.7725 |
| LDA | 0.5792 | 0.5776 | 0.6945 |
| Vanilla NN | 0.7816 | 0.7847 | 0.9161 |
| Add | 0.8262 | 0.8288 | **0.9373** |
| Concat | **0.8275** | **0.8299** | 0.9365 |

**References**

1 Sun, F.-Y., Hoffmann, J., Verma, V. & Tang, J. Infograph: Unsupervised and semi-supervised graph-level representation learning via mutual information maximization. *arXiv preprint arXiv:1908.01000* (2019).

2 Kim, D., Paggi, J. M., Park, C., Bennett, C. & Salzberg, S. L. Graph-based genome alignment and genotyping with HISAT2 and HISAT-genotype. *Nat Biotechnol* **37**, 907–915 (2019). <https://doi.org/10.1038/s41587-019-0201-4>

3 Love, M. I., Huber, W. & Anders, S. Moderated estimation of fold change and dispersion for RNA-seq data with DESeq2. *Genome Biol* **15**, 550 (2014). <https://doi.org/10.1186/s13059-014-0550-8>

4 Wu, T. *et al.* clusterProfiler 4.0: A universal enrichment tool for interpreting omics data. *Innovation (Camb)* **2**, 100141 (2021). <https://doi.org/10.1016/j.xinn.2021.100141>
